# Involvement of miR-15a in G0/G1 Phase Cell Cycle Arrest Induced by Porcine Circovirus Type 2 Replication

**DOI:** 10.1038/srep27917

**Published:** 2016-06-15

**Authors:** Rong Quan, Li Wei, Shanshan Zhu, Jing Wang, Yongchang Cao, Chunyi Xue, Xu Yan, Jue Liu

**Affiliations:** 1 Beijing Key Laboratory for Prevention and Control of Infectious Diseases in Livestock and Poultry, Institute of Animal Husbandry and Veterinary Medicine, Beijing Academy of Agriculture and Forestry Sciences, No. 9 Shuguang Garden Middle Road, Haidian District, Beijing 100097, P. R. China; 2 Life Sciences School, Sun Yat-sen University, Guangzhou Higher Education 11 Mega Center, Guangzhou 510006, P. R. China

## Abstract

Many viruses exploit the host cell division cycle to favour their own growth. Here we demonstrated that porcine circovirus type 2 (PCV2), which is a major causative agent of an emerging and important swine disease complex, PCV2-associated diseases, caused G0/G1 cell cycle arrest through degradation of cyclin D1 and E followed by reduction of retinoblastoma phosphorylation in synchronized PCV2-infected cells dependent upon virus replication. This induction of G0/G1 cell cycle arrest promoted PCV2 replication as evidenced by increased viral protein expression and progeny virus production in the synchronized PCV2-infected cells. To delineate a mechanism of miRNAs in regulating PCV2-induced G0/G1 cell cycle arrest, we determined expression levels of some relevant miRNAs and found that only miR-15a but not miR-16, miR-21, and miR-34a was significantly changed in the PCV2-infected cells. We further demonstrated that upregulation of miR-15a promoted PCV2-induced G0/G1 cell cycle arrest via mediating cyclins D1 and E degradation, in which involves PCV2 growth. These results reveal that G0/G1 cell cycle arrest induced by PCV2 may provide favourable conditions for viral protein expression and progeny production and that miR-15a is implicated in PCV2-induced cell cycle control, thereby contributing to efficient viral replication.

Porcine circovirus type 2 (PCV2), a genus Circovirus of the family Circoviridae^1^, has been shown to associate with a variety of clinical disorders, including postweaning multisystemic wasting syndrome (PMWS)^2^,^3^, porcine dermatitis and nephropathy syndrome, reproductive failure, necrotizing tracheitis, congenital tremors as well as fetal myocarditis^4^,^5^,^6^,^7^. In addition, immunosuppression mediated by PCV2 infection leads to the susceptibility of PCV2-infected pigs to other infectious agents and the reduced ability of immune response to vaccinations^7^. PCV2-associated disease (PCVAD) now affects most pig-producing regions, leading to huge economic losses to the pig industry worldwide.

Up to date, five open reading frames (ORF) have been recognized for PCV2. ORF1 encodes a non-structural, Rep protein which is essential for viral replication^8^. ORF2 encodes the only structural capsid (Cap) protein conferring host-protective function^9^,^10^. Besides the Rep and Cap proteins, ORF3 and ORF4 proteins, are considered to be involved in viral pathogenesis via apoptotic and anti-apoptotic functions during PCV2 infection, respectively^11^,^12^,^13^; ORF5 protein is considered to prolong S-phase of cell cycle and induce NF-κB activation^14^.

Increasing research evidence indicates that PCV2 infection regulates many host cellular signals and pathways, such as nuclear transcription factor kappa B (NF-κB)^15^, extracellular signal-regulated kinase^16^, c-Jun NH2-terminal kinases (JNK1/2) and p38 mitogen-activated protein kinase (MAPK)^17^, and phosphatidylinositol 3-kinase (PI3K)/Akt^18^, which contributed to PCV2 replication and PCV2-mediated apoptotic responses. Many viruses exploit the host cell division cycle to favour their own growth. The cell cycle consists of DNA replication (S phase), mitosis (M), and cytokinesis, separated by two gaps (G1 and G2). Quiescent cells are referred as being in G0 phase. Cyclin D-Cdk (cyclin-dependent kinase) 4/6 complexes and phosphorylation of the downstream retinoblastoma (Rb) protein initiate and regulate G1-phase progression, and cyclin E-Cdk2 activity is important in the G1/S transition and DNA replication^19^. Cellular Cdk inhibitors (CKIs) are also involved in G1-phase progression. Increasingly, some viruses or their proteins, including herpesviruses^20^,^21^, murine hepatitis virus (MHV) and its nonstructural protein p28^22^,^23^, severe acute respiratory syndrome coronvirus (SARS-CoV) proteins^24^,^25^,^26^, influenza A virus and its NS1 protein^27^,^28^, human respiratory syncytial virus^29^, and murine norovirus (MNV)^30^, are able to induce cell cycle arrest in the G0/G1 phase and induction of G0/G1 cell cycle arrest was exploited by these viruses for their efficient replication. In a previous report, PCV2-induced apoptosis has been shown to require activation of p53^31^. As a multifunctional transcription factor, p53 has been considered to play a role in both induction of apoptosis and regulation of cell cycle^32^,^33^. Furthermore, cross talk has been proposed between induction of apoptosis and cell cycle control^34^. Thus, it prompted us to investigate whether PCV2 infection affects the cell cycle progression, which facilitated for virus growth.

MicroRNAs (miRNAs) are a novel class of small regulatory RNA molecules at the post-transcriptional level and involved in varieties of biological processes, including cell fate specification, proliferation and differentiation, apoptotic responses^35^. Notably, miRNAs may play critical roles in gene regulation network of the cell cycle control machinery. Increasing research data has shown that some host miRNAs are implicated in regulation of cell cycle progression. miR-15a/16 family has been shown to regulate the G0/G1 cell cycle progression by targeting cyclins D1 (CCND1) and E (CCNE)^36^,^37^. Also, miR-16, which possesses a spectrum of potential targets, co-ordinately regulated different mRNA targets, including CDK6, CARD10, CDC27, C10orf46, as well as G1-related cyclins, acted in concert to control cell cycle progression^38^,^39^. miR-21 has been shown to play an important role in regulating cell cycle via targeting Cdc25a, which participates in G_1_-to-S transition^40^,^41^. miR-34a involved induction of cell cycle arrest by downregulating CCND1 and CDK6 expression^42^. However, whether host miRNA induced by PCV2 infection involved PCV2-mediated cell cycle arrest and contributed to virus replication is not clear.

In the present study, we examined whether PCV2 infection affects the cell cycle progression and found that PCV2 replication induces cell cycle arrest in G0/G1 phase, which facilitates to producing favourable conditions for viral protein expression and virus production. In addition, we determined expression of some host miRNAs (miR-15a/16, miR-21, and miR-34a), which are related to regulation of cell cycle control, and found that only miR-15a was significantly changed. This upregulation of miR-15a expression may contribute to PCV2-induced G0/G1 cell cycle arrest via decreasing expression of cyclins D1 and E to block cell cycle progression, thereby facilitating for virus replication.

## Results and Discussion

### Cell cycle arrest induced by PCV2 infection occurred in G0/G1 phase depends upon viral replication

**Figure 1:**
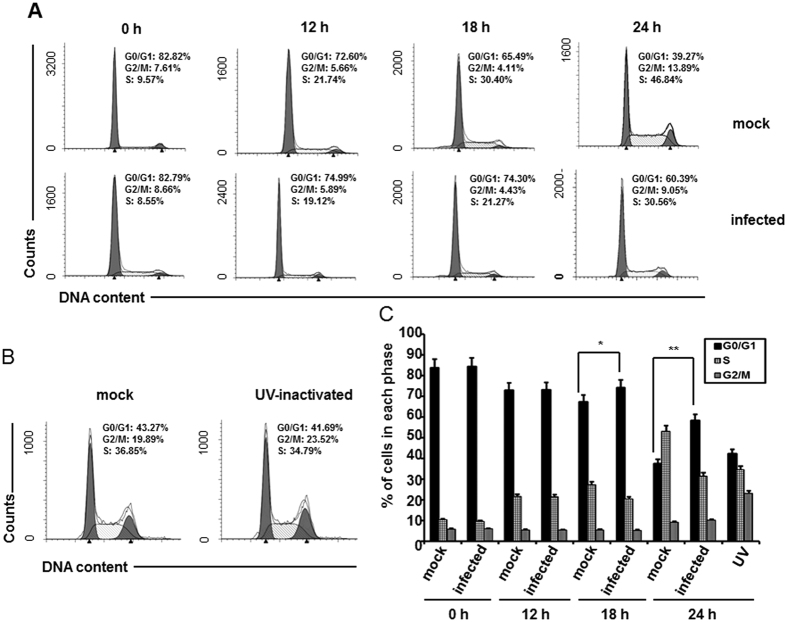
PCV2 infection induces G0/G1 phase cell cycle arrest dependent on viral replication. Synchronized PK15 cells were mock infected (mock) or infected with PCV2 (infected) or UV-inactivated PCV2 (UV inactivated) at an MOI of 1. At the indicated time points postinfection, cells were collected, stained with PI, and analyzed by FACS analysis. (**A,B**) The cell cycle profile shown was obtained from one of three independent experiments. The data were analyzed by the ModFit program from one of three independent experiments. (**C**) The histograms showed the percentage of cells in each phase of the cell cycles. The results are shown as means ± standard deviation (SD) for three independent experiments. *P < 0.05; **P < 0.01.

Research evidence has shown that activation of p53 involved PCV2-induced apoptotic response^31^, thus, we want to determine whether PCV2 infection of PK15 cells regulated cell cycle progression. Synchronized PK15 cells maintained in minimal essential medium (MEM) containing no serum for 48 h were infected with PCV2 strain BJW^36^ at a multiplicity of infection (MOI) of 1 50% tissue culture infective dose units (TCID_50_) per cell and incubated with MEM supplemented with 5% fetal bovine serum (FBS). At the indicated time points postinfection, nuclear DNA content was measured using propidium iodide (PI) staining and fluorescence-activated cell sorting (FACS) analysis. As shown in Fig. 1A, we observed an obvious accumulation of cells in G0/G1 phase in the synchronized PCV2-infected cells at 18 and 24 h postinfection. To examine whether cell cycle arrest induced by PCV2 is associated with virus replication, we used an UV light-irradiated PCV2 (equivalent to an MOI of 1) to infect synchronized cells. The G0/G1 cell cycle arrest was not detectable in cells infected with UV-treated PCV2 or in mock-infected cells (Fig. 1B). The histograms were further analyzed quantitatively by a curve-fitting program to determine the percentage of cells in each of the G0/G1, S, and G2/M phase (Fig. 1C). Higher percentages of PCV2-infected cells were in the G0/G1 phase at 18 and 24 h postinfection, at which time about 10 and 55% larger G0/G1-phase populations were present in the PCV2-infected cells than in the mock-infected cells, respectively. The cell population in G0/G1 phase after infection with UV-treated PCV2 decreased significantly as compared with that in the PCV2-infected cells, but which is comparable to that in mock-infected cells (Fig. 1C). Together, these data suggest that PCV2 is capable of inducing a G0/G1 phase arrest in the infected cells dependent upon virus replication.

### Involvement of key molecules in regulating PCV2-induced cell cycle arrest

**Figure 2:**
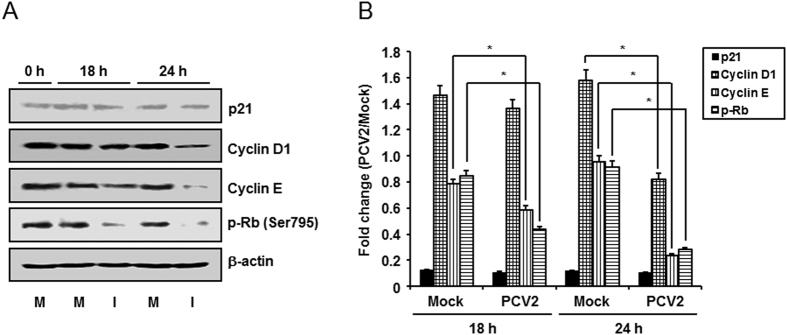
Effect of PCV2 infection on the expression levels of total cellular cell cycle regulatory proteins. **(A)** Synchronized PK15 cells were mock infected (M) or infected with PCV2 (I) at an MOI of 1 and then collected at the indicated time points after infection. Cell lysates were resolved by sodium dodecyl sulphate-polyacrylamide gel electrophoresis (SDS-PAGE), transferred to nitrocellular membranes, and immunoblotted with antibodies to cyclin D1, cyclin E, regulatory factor p21, and phosphorylated Rb (Ser795). Equal protein loads were verified with β-actin blots. p-, phosphorylated. (**B**) Amounts of the cell cycle regulator proteins in panel A were quantified by densitometric analysis and normalized against β-actin. Bars indicate the ratio of the cell cycle regulatory proteins in PCV2-infected samples to those in mock- infected samples. The results are presented as the means ± SD for three independent experiments. *P < 0.05.

Many host molecules participate in regulation of the G1/S transition, such as cyclin-Cdk complexes, pRb, and CKI molecules^43^. In order to further confirm the key molecules responsible for PCV2-induced cell cycle arrest, synchronized PCV2-infected cells were assayed for expression of several host G1/S transition proteins by Western blotting at 18 and 24 h postinfection. As shown in Fig. 2A, there were decreases of cyclins D1 and E in the PCV2-infected cells as compared to mock-infected cells. In contrast, no significant difference was observed in the amounts of p21 between mock-infected and PCV2-infected cells at the indicated time points postinfection (Fig. 2A), indicating that PCV2-induced G0/G1 cell cycle arrest did not involve the activation of p21, as observed for murine coronavirus^22^. As a downstream target of cyclins D1 and E, the level of phosphorylated retinoblastoma (p-Rb) was significantly reduced after infection (Fig. 2A). Densitometric analysis further confirmed that the amounts of cyclins D1 and E, as well as p-Rb were markedly smaller in the PCV2-infected cells than in mock-infected cells at 18 and 24 h postinfection (Fig. 2B). These results indicate that PCV2 infection limited the expression of cyclins D1 and E followed by inhibiting Rb phosphorylation, and in turn leading to a block in the G0/G1 to S phase of the cell cycle. Similar observations were reported in other viruses, such as measles virus^44^, herpes simplex virus type 1^20^,^45^, murine coronavirus^22^, influenza A virus^27^, and MNV^30^. This indicated that a reduction in the amounts of G1 cyclins may be a common mechanism by which some viruses including PCV2 block G1 cell cycle progression.

### G0/G1-phase-synchronized cells promote PCV2 viral replication

**Figure 3:**
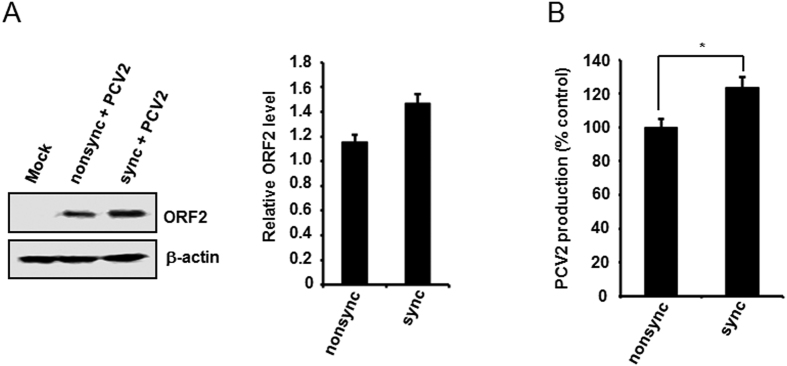
G0/G1 phase cell cycle accumulation promotes PCV2 growth. (**A**) Total lysates of synchronized or nonsynchronized PCV2-infected PK15 cells at 24 h postinfection were assayed for the amount of viral protein synthesis by Western blot analysis against probe for ORF2 protein. β-actin expression was monitored as a loading control. The bands of ORF2 protein were quantified using densitometric analysis and plotted after normalization against β-actin. The histogram shows means ± SD for three independent experiments. nonsync, nonsynchronized; sync, synchronized. (**B**) Supernatants of synchronized or nonsynchronized PCV2-infected PK15 cells at 24 h postinfection were inoculated on monolayers of PK15 cells and incubated for 72 h. Virus productions were determined using the IFA method under a fluorescence microscopy. The values represented are the means of the results for three independent experiments. *P < 0.05.

Increasing research data has shown that many viruses are capable of exploiting G0/G1 cell cycle arrest for their efficient replication^22^,^27^,^30^. To investigate whether the cell cycle arrest in G0/G1 phase induced by PCV2 infection was advantageous for virus replication, total lysates from synchronized or non-synchronized PCV2-infected cells were harvested individually at 24 h postinfection. The non-synchronized cells were maintained in normal MEM containing serum. As shown in Fig. 3A, the expression level of viral ORF2 protein in total cell lysates from synchronized cells was higher than that in non-synchronized cells as determined by Western blotting and further densitometric analysis. In order to determine whether the increase in viral protein expression would facilitate progeny virus production, supernatants from infected cells were examined for virus production by an indirect immunofluorescence assay (IFA). As expected, more PCV2-positive signals were seen in the synchronized cells as compared to the non-synchronized cells (data not shown). Evaluation of PCV2-positive cells showed that PCV2 virus production was increased by about 23% after synchronization treatment (Fig. 3B). This is consistent with the pattern of viral protein expression levels (Fig. 3A). The results indicated that PCV2 can exploit the G0/G1 phase of the cell cycle for its own growth.

### miR-15a was upregulated in PCV2-infected cells

**Figure 4:**
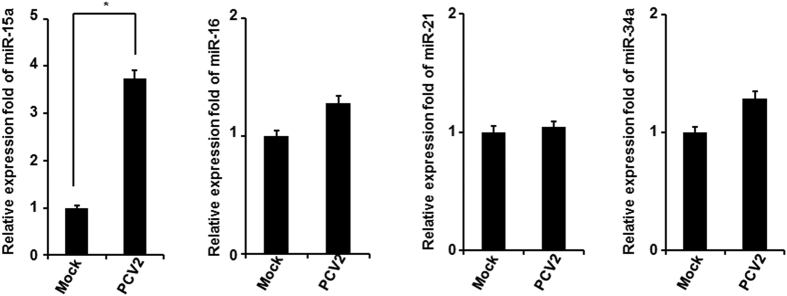
Upregulation of miR-15a expression in PCV2-infected PK15 cells. Synchronized PK15 cells were mock infected or infected with PCV2 at an MOI of 1. At 24 h postinfection, expression levels of miR-15a and miR-16 as well as miR-21 and miR-34a were assayed by qRT-PCR. U6 was used for normalization of miRNA qRT-PCR. The values represent the means of results from three independent experiments. *P < 0.05.

The above results indicated that the G0/G1 cell cycle arrest in PCV2-infected cells was mediated by decrease of cyclins D1 and E. Research data has shown that miR-15a/16 family share many common targets including cyclins D1 and E, regulating the G0/G1 cell cycle progression^36^,^37^. This prompted us to analyse whether these two host miRNAs are co-regulated after PCV2 infection and they involved G0/G1 cell cycle arrest induced by PCV2 replication. Thus, we determined the expression levels of both miRNAs in the synchronized PCV2-infected PK15 cells by real-time RT-PCR. As shown in Fig. 4, significant upregulation of miRNA-15a (3.7 folds) was observed in the synchronized cells at 24 h postinfection as compared to that in mock-infected cells. In contrast, miR-16 expression levels were not obviously changed after PCV2 infection, comparable to that in mock-infected cells (Fig. 4). miR-21 and miR-34a have been also shown to regulate the cell cycle progression by targeting relevant G1-to-S transit proteins. Therefore, we further determined the expression levels of miR-21 and miR-34a in the synchronized PCV2-infected PK15 cells and found that no obvious changes were observed as compared to that in the mock-infected cells (Fig. 4). These results demonstrated that PCV2 infection mediated upregulation of only miR-15a expression, which prompted us to speculate that miR-15a may involve in PCV2-induced G0/G1 cell cycle arrest.

### miR-15a downregulated cell cycle regulators CCND1 and CCNE expression and promoted G0/G1 cycle arrest in PCV2-infected cells

**Figure 5:**
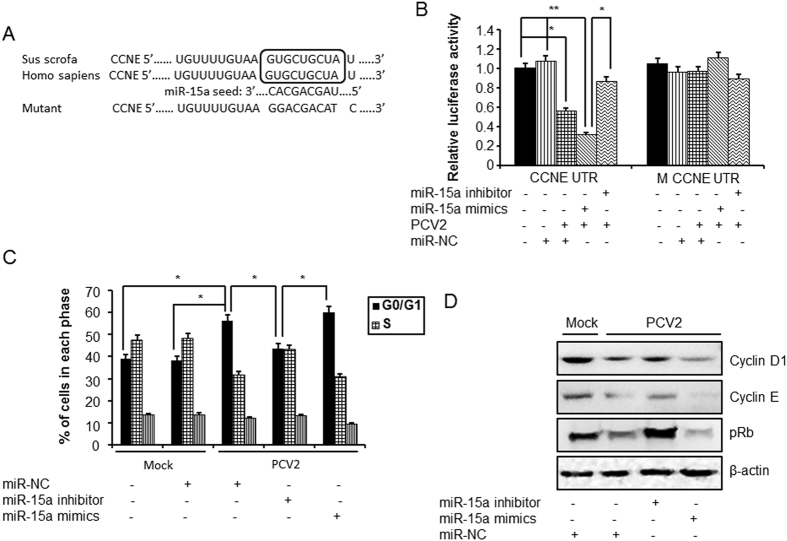
miR-15a regulates PCV2-induced G0/G1 phase cell cycle arrest. (**A**) Predicted binding sequence is indicated between the seed sequence of miR-15a and its target sequence CCNE 3′UTR. (**B**) pGL3 Luciferase Reporter Vector containing porcine CCNE 3′UTR were co-transfected into synchronized PK15 cells with pRL-TK 24 hours after transfection of miR-15a inhibitors, miR-15a mimics and controls. 12 h after first transfection, the cells were infected with PCV2. 24 hours after the report plasmids tranfection, the cells were assayed using a dual luciferase assay. Firefly luciferase values were normalized to Renilla luciferase values and plotted as relative luciferase activity (means ± SD, n = 3). *Significantly different from wild-type reporter (p < 0.05). (**C**) Synchronized PK15 cells infected with PCV2 (24 h) after transfection with the miR-15a inhibitor or NC were assayed by FACS analysis. The histograms showed the percentage of cells in each phase of the cell cycles. The values are shown as the means of results from three independent experiments. *P < 0.05. (**D**) Total lysates from synchronized cells-infected with PCV2 (24 h) after transfection with the miR-15a inhibitor were subject to Western blotting for cyclin D1, cyclin E, and phosphorylated Rb (Ser795). β-actin was probed as the loading control. p-, phosphorylated.

To validate the possibility that miR-15a may target key molecules related to PCV2-induced G0/G1 cell cycle arrest, we used RNA hybrid 2.2 to analyse and found that miR-15a seed sequence possesses the highest score of probability for targeting cyclin E (CCNE) 3′ UTR (Fig. 5A). The public miRNA database (RNAhybrid 2.2) predicted that cyclin E might be a target for miR-15a, with a highly conserved binding site occurring at the 3′ UTR of Sus scrofa cyclin E (position 1786 to 1794) (Sus scrofa cyclin E, XM_003127005) mRNA for miR-15a (NR_035364). We have not found the complete 3′ UTR of Sus scrofa cyclin D1 (CCND1) and cannot predict whether Sus scrofa CCND1 possesses a highly conserved binding site, but the binding site to miR-15a was found to occur at other species such as the 3′ UTR of Gallus gallus cyclin D1 (position 1816–1823), and this binding site is highly homology to that in the 3′ UTR of sus scrofa cyclin E (position 1786 to 1794). Therefore, dual luciferase reporter assay was performed to only investigate whether miR-15a directly target the 3′ UTR of cyclin E mRNAs. As shown in Fig. 5B, a significant decrease in the relative luciferase activity was observed in the PCV2-infected cells after transfected with miR-15a mimics as compared to that in the negative control (miR-NC). The relative luciferase activity in the PCV2-infected miR-15a inhibitor-treated cells was higher than that in the PCV2 alone-infected cells. In addition, we used pGL3-control-CCNE (mutant) plasmid (Fig. 5A) to transfect PCV2-infected cells and found that the relative luciferase activity of the PCV2-infected cells was not obviously changed, regardless of co-transfection with miR-NC, miR-15a inhibitor, or miR-15a mimics, as compared to that in mock-infected miR-NC-treated cells (Fig. 5B). Collectively, these results suggested that miR-15a targets cyclin E mRNA at 3′ UTR.

To access the role of miR-15a expression in PCV2-induced blockage of G0/G1 cycle progression, the cells were transfected with miR-15a inhibitor before PCV2 infection for 24 h and the cell cycle was analyzed by flow cytometry. As shown in Fig. 5C, inhibition of miR-15a expression can rescue PCV2-induced G_0_/G_1_ phase cell cycle block, at which time about 56% and 43% G0/G1-phase populations for PCV2-infected and PCV2-infected miR-15a inhibitor-treated cells, respectively, as compared to that in the mock-infected cells. G0/G1-phase populations were slightly increased in the PCV2-infected cells after treatment with miR-15a mimics (Fig. 5C). We further used Western blotting to determine the expression of cyclins D1 and E in the PCV2-infected miR-15a inhibitor-treated cells and found that decrease of cyclins D1 and E as well as reduction in the level of Rb phosphorylation were also recovered in the PCV2-infected cells after miR-15a inhibitor treatment (Fig. 5D). However, treatment of miR-15a mimics obviously reduced the expressions of cyclins D1 and E as well as p-Rb in the PCV2-infected cells (Fig. 5D). These results further demonstrated that overexpression of miR-15a induced by PCV2 decreased cyclins D1 and E expression followed by reduction of Rb phosphorylation, thus leading to the blockage of cell cycle in G0/G1 phase in PK15 cells.

### Upregulation of miR-15a is beneficial for PCV2 growth

**Figure 6:**
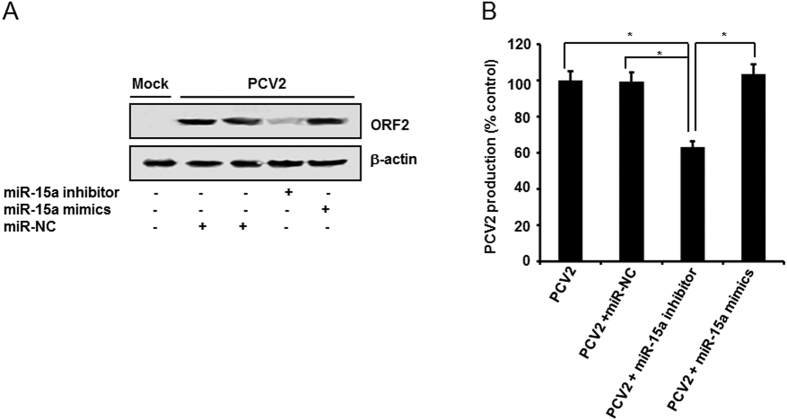
miR-15a promotes PCV2 growth. (**A**) Synchronized PK15 cells infected with PCV2 (24 h) after transfection with the miRNA-15a inhibitor were assayed for viral ORF2 protein expression by Western blot analysis. β-actin was probed as the loading control. (**B**) Synchronized PK15 cells infected with PCV2 (24 h) after transfection with the miRNA-15a inhibitor or mimics were inoculated on monolayers of PK15 cells and virus production was assayed by the IFA method. The values represent the means of results from three independent experiments. *P < 0.05.

The above data demonstrated that expression of miR-15a is upregulated in the PCV2-infected PK15 cells and that miR-15a regulated expression of cyclins D1 and E, which are required for promoting cells from G1 to S phase. Thus, we wanted further to assess the effects of miR-15a on PCV2 replication. PK15 cells were transfected with a specific miR-15a inhibitor or control miR-NC followed by infection with PCV2 (MOI = 1) for 24 h. Western blot analysis showed that the expression levels of viral ORF2 protein were significantly decreased after treatment with miR-15a inhibitor (Fig. 6A). For miR-15a mimics treatment, expression of ORF2 protein was slightly increased when compared to that in the PCV2 alone-infected cells (Fig. 6A). As expected, no obvious changes were observed in the expression levels of ORF2 protein in PCV2-infected miR-NC-treated cells (Fig. 6A). The supernatants from PCV2-infected miR-15a inhibitor-treated cells were further collected for determination of virus production. As shown in Fig. 6B, evaluation of PCV2-positive cells showed that PCV2 virus production after miR-15a inhibition was significantly decreased (p < 0.05) as compared to that in the PCV2-infected miR-NC-treated cells. Treatment of miR-15a mimics slightly increased PCV2 virus production when compared to that in the PCV2 alone-infected cells (Fig. 6B). These results, together with the data shown in Fig. 5C,D, indicated that upregulation of miR-15a is beneficial for PCV2 growth and that contribution of miR-15a to PCV2-induced G0/G1 phase in PK15 cells.

G0/G1 cell cycle arrest has been reported for infection of many viruses. MHV infection was reported to inhibit host cellular DNA synthesis and block G0/G1 phase progression in 17CL-1 and DBT cells through cyclin D2 and cyclin E degradation^22^, in which nonstructural protein p28 might be responsible for MHV to induce G0/G1 cell cycle arrest^23^. Cyclin D3 decrease was observed in measles virus (MV)-infected cells and may contribute to MV-induced G1 cell cycle arrest^44^. Infection of avian influenza virus was reported to induce accumulation of cells in G0/G1 phase in the infected cells through cyclin D1 and cyclin E degradation^27^, in which NS1 protein interacted with the RhoA/Rb pathway^28^. This G0/G1 arrest was demonstrated to promote influenza A virus replication, as evidenced by increased viral protein expression and virus production in G0/G1 phase-arrested cells^27^. MNV infection was shown to induce an arrest in G0/G1 phase by inhibiting G1/S transition, providing a more favourable environment for its own replication^30^. During viral infection, degradation of cyclin E will decrease cyclin E-Cdk2 activity, while degradation of cyclin D will decrease cyclin D-Cdk4/6 activity. Phosphorylation of Rb, which is regulated primarily by complexes of cyclin D-Cdk4/6 and later by cyclin E-Cdk2 activity, is a critical step in the G1 to S phase transition. Similar to these viruses, infection of PCV2 leads to the decreased expression of both cyclin D1 and cyclin E followed by down-regulation of the Rb phosphorylation, thereby blocking cell cycle progression in G0/G1 phase. Furthermore, PCV2 infection-induced G0/G1 arrest helped to facilitate viral replication, this might be associated with better adaptation of PCV2 infection to these conditions.

miRNAs contribute to cell cycle control via targeting a variety of key molecules of the cell cycle machinery for the coordinated regulation of gene expression^46^,^47^. Increasing research data have shown that many viruses exploit host cellular miRNAs to modify cell cycle control for viral replication. Decreased expression of miR-122 during HBV infection was shown to lead to upregulation of its target cyclin G1 followed by modulating p53 activity, thereby possibly contributing to viral replication and persistence^48^. Human cytomegalovirus was reported to inhibit expression of miR-21 targeting Cdc25a followed by promoting cells to enter G1/S transition, thereby benefiting viral replication^49^. Upregulation of let-7c expression after HIV-1 infection in T-lymphocytes and HeLa-CCR5 cell lines was demonstrated to promote cell cycle progression via targeting and downregulating p21 protein, a negative regulator of the G1/S transition, which facilitating to viral replication^50^. In the present study, we found that only miR-15a but not other miRNAs including miR-16, miR-21, and miR-34a, exhibited significant upregulation in the synchronized PCV2-infected PK15 cells. After predicating the putative targets of miR-15a, we further found CCNE (cyclin E) as a direct target of miR-15a in PK15 cells through luciferase assay. Although there is not an direct evidence that CCND1 (cyclin D1) might be also a target of miR-15a in PK15 cells due to the absence of the complete 3′ UTR of Sus scrofa cyclin D1 available, we found that limitation of miR-15a expression by treatment with miR-15a inhibitor promoted cyclins D1 and E protein expression in synchronized PCV2-infected PK15 cells, in which miR-15a-induced cell cycle arrest can be restored after inhibition of miR-15a expression. This might implicate that miR-15a also regulates expression of cyclin D1 protein via direct interaction with target sequence located at the 3′ UTR of Sus scrofa cyclin D1, as observed for cyclin E. In addition, we found that miR-15a promoted more efficient PCV2 replication as confirmed by decreased viral protein expression and virus production in the PCV2-infected cells after inhibition of miR-15a expression. Given that CCND1/CCNE and miR-15a are inversely regulated during PCV2 infection, we suggested that involvement of miR-15a in augmenting PCV2 replication is mainly modulated by its ability to downregulate expression of CCND1 and CCNE. Overall, these results showed that PCV2 upregulates miR-15a as a means to decrease CCND1 and CCNE levels, which may benefit viral replication by blocking G0/G1 cell cycle progression.

In conclusion, these results demonstrated that PCV2 replication blocks cell cycle progression at the G0/G1 phase, which produces favourable conditions for viral protein expression and virus production. PCV2-induced G0/G1 cell cycle arrest may be regulated by host miRNA-15a via mediating cyclins D1 and E degradation, in which enhances PCV2 growth. Knowledge of the role of miRNA-15a in regulating cell cycle control will provide important information for understanding the molecular mechanism of PCV2 infection.

## Methods

### Cell culture and virus infection

PK15 cells were cultured in minimal essential medium (MEM) supplemented with 5% fetal bovine serum (FBS), 5% L-glutamine, and 1% penicillin-streptomycin at 37 °C and 5% CO_2_. Subconfluent cultures of PK15 cells were starved without serum addition for 48 h for synchronization. Synchronized cells were mock infected or infected with PCV2 strain BJW^11^ at a multiplicity of infection (MOI) of one 50% tissue culture infective dose units (TCID_50_) per cell. After 1 h of virus adsorption, cells were treated with medium containing 5% FBS and harvested at the indicated times post-infection for cell cycle analysis and Western blotting.

### Antibodies

Rabbit, goat, or mouse antibodies against cyclin D1, cyclin E, phosphorylated-Rb (p-Rb) (Ser795), p21, β-actin were purchased from Santa Cruz Biotechnology. Horseradish peroxidase (HRP)-conjugated secondary antibodies were purchased from Sigma. Fluorescein isothiocyanate (FITC)-conjugated secondary antibodies were purchased from Dako.

### Cell cycle analysis

Flow cytometry was used to analyze cell cycle as described previously^16^. In brief, adherent PK15 cells after mock infected or infected with PCV2 for 0, 12, 18 and 24 h were harvested by trysinization, washed with phosphate-buffered saline (PBS), and pelleted by centrifugation. UV-inactivated PCV2 was prepared and used as described previously^18^. The cells were fixed in 80% ethanol overnight at 4 °C and stained for nuclear DNA content with 50 μg/ml propidium iodide (PI) and 1 mg/ml RNase A at room temperature (RT) for 15 min. Determination of PI-stained cells was performed using fluorescence-activated cell sorting (FACS) on BD FACS Canto™ II. At least 30,000 cells were counted for each sample. Data was analysed by using ModFit LT, Version 2.0 (Verity Software House).

### miRNA quantification

Total RNA was extracted from synchronized PK15 cells after PCV2 infection using Trizol Reagent (Invitrogen). Quantitative reverse transcriptase-polymerase chain reaction (qRT-PCR) was used to determine the expression levels of miRNAs according to the protocol of miRcute miRNA cDNA kit (Tiangen Biotech, Co., LTD, Beijing). Briefly, the extracted RNA was polyadenylated and the first strand complementary DNA was synthesized using oligo (dT). U6 snRNA was used for normalization. The primers of miRNAs amplification include the universal primer and miR specific forward primers (ssc-miR-15a F: 5′-TAGCAGCACATAATGG-3′, ssc-miR-16 F: 5′-TAGCAGCACGTAAATATTGGCG-3′, ssc-miR-21 F: 5′-TAGCTTATCAGACTGATGTTG-3′, and ssc-miR-34a F: 5′-TGGCAGTGTCTTAGCTGGT-3′). U6 snRNA F: 5′-CTCGCTTCGGCAGCACA-3′, U6 snRNA R: 5′-AACGCTTCACGAATTTGCGT-3′. Relative expression was analyzed using the ΔΔCt method.

### Plasmid construction

The entire 3′ UTR of target gene Sus Scrofa cyclin E (XM_003127005) were amplified from porcine mRNA by RT-PCR, and then cloned into downstream of the luciferase open reading frame (ORF) in the vector pGL3-control (Invitrogen). The primers (CCNE F: 5′-GCTCTAGA CTGCAGCAGAGGCCTGCAT-3′ and CCNE R: 5′-GCTCTAGACCCTCAACGAACCCATACATAC-3′, and CCNE MF: 5′- GGACGACATCGTCTCTCCGTTTTTTAATAAAGATGACACTGTC-3′ and CCNE MR: 5′-ACGGAGAGACGATGTCGTCCTTACAAAACAATAGTTC CNE-3′) were used to create the plasmids pGL3-control-CCNE-3′ UTR (wild-type) and pGL3-control-CCNE M-3′ UTR (mutant), respectively. The single restriction enzyme cutting site is XbaI. The vector pRL-TK (Promega) was also used as the co-transfection vector for dual luciferase analysis experiments.

### Transfection and luciferase assay

PK15 cells were grown and starved for 48 h in 96-well plate before transfection. To investigate miR-15a function in PCV2-infected PK15 cells, we purchase miRNA-15a mimics and inhibitor from GenePharma Co., Ltd., Shanghai, China. The synchronized PK15 cells were transfected with miRNA-15a mimics/inhibitor or negative control (miRNA-NC) (GenePharma, sequence: CAGUACUUUUGUGUAGUACAA) at a final concentration of 30–40 nmol/l using Lipofectamine 2000 (Invitrogen) following the manufacturer’s procedure. Twelve hours after transfection, PK15 cells were transfected with 500 ng of pGL3-control-CCNE-3′ UTR or pGL3-control-CCNE M-3′ UTR plasmid and 25 ng of pRL-TK, and twenty-four hours later cells were washed and mock infected or infected with PCV2 strain BJW at an MOI of 1, and maintained in MEM supplemented with 5% FBS for incubation. Then, cells were harvested 24 h after infection and analyzed for luciferase activity and Western blotting. Luciferase assays were performed using a Dual-Luciferase Reporter Assay System according to the manufacturer’s instruction (Promega). The luminescence intensity of firefly luciferase was normalized to that of Renilla luciferase, and the results were expressed as the relative luciferase activity.

### Determination of virus production

An indirect immunofluorescence assay (IFA) was used to determine PCV2 progeny production as described elsewhere^18^ with a minor modification. Briefly, PK15 monolayer cells infected with supernatants of synchronized or non-synchronized PCV2-infected cells were incubated for 72 h. After fixation with 4% paraformaldehyde-PBS, the cells were incubated with rabbit anti-ORF2 antibody diluted in 1% skim powder-PBS at RT for 2 h followed by fluorescein isothiocyanate (FITC)-conjugated anti-rabbit immunoglobulin G (Sigma) at RT for 1 h. The cells were then examined under fluorescence microscopy and the PCV2-positive cells were counted in random six fields of view.

### Western blotting

Whole PK15 cell lysate extracts at different time points after PCV2 infection were prepared with a nuclear extract kit (Active Motif) following the manufacturer’s protocol. The cell lysate extracts were diluted in 2× sample buffer and boiled for 5 min. 20 μg of each protein extract was subjected to 12% sodium dodecyl sulfate-polyacrylamide gel electrophoresis (SDS-PAGE) and transferred to nitrocellulose membranes. The membranes were blocked in TBST blocking buffer (20 mM Tris-HCl [pH 7.4], 150 mM NaCl, 0.1% Tween 20) containing 5% dry skim milk powder at RT for 2 h to prevent nonspecific binding. The membranes were then incubated with the antibodies raised against cyclin D1, cyclin E, p-Rb (Ser795), p21, ORF2, and β-actin at RT for 2 h. After three washes with TBST buffer, the membranes were incubated with horseradish peroxidase-conjugated secondary antibodies diluted in blocking buffer at RT for 1 h. Immunoreactive bands were detected by enhanced Chemiluminescence system (Kodak Image Station 4000R).

### Statistical analyses

The averaged values from three independent experiments were analyzed by one-way analysis of variance and Student’s *t* test. Results were represented as means ± the standard deviation (SD) of the means. Differences between groups were considered to be significant when P values were <0.05.

## Additional Information

**How to cite this article**: Quan, R. *et al.* Involvement of miR-15a in G0/G1 Phase Cell Cycle Arrest Induced by Porcine Circovirus Type 2 Replication. *Sci. Rep.* **6**, 27917; doi: 10.1038/srep27917 (2016).
